# Supplementary material for: Event-related potentials reveal limited readiness to access phonetic details during word processing in dogs
Source: R Soc Open Sci. 2020 Dec 9;7(12):200851. doi: 10.1098/rsos.200851 (PMC7813267; doi:10.1098/rsos.200851)
Supplement: Supplementary Material to Magyari et al., Event-related potentials reveal limited readiness to access phonetic details during word processing in dogs [file rsos200851supp1.pdf]

Supplementary material to Magyari et al., Event-related potentials reveal limited readiness to access phonetic details during word processing in dogs

Supplemental Methods

Stimuli

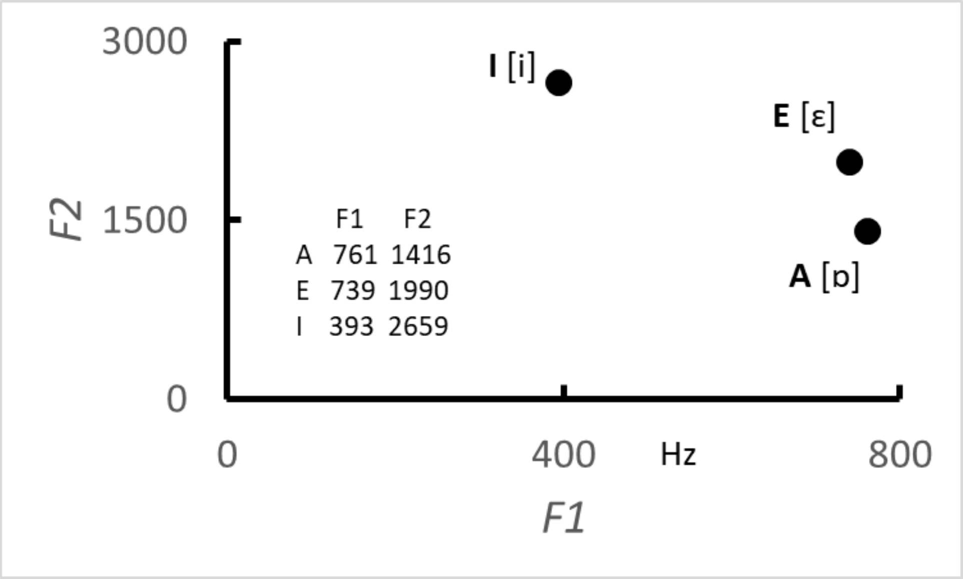

Fig. S1. Vowel space of the vowels in the first syllable of all words used as stimuli.

WORDS	SIMILAR	Frequency of initial consonants (/million words)	NONSENSE	Frequency of initial consonants (/million words)
Fekszik	Fakszik	32336.44	Matszer	71792.51
Marad	Merad	71792.51	Hefegy	46673.01
Gyere	Gyare	4058.86	Dime	10953.02
Mehetsz	Mihetsz	71792.51	Rekaksz	14845.30

Table S1. Frequency of words starting with the initial consonants of the stimuli. Frequency data is based on the Hungarian National Corpus (1) which contains 187.6 million words.

Supplemental Results and Discussion

Amplitude-based data cleaning with 20 Hz low-pass filter

In order to better visualize a general shape of the ERP results, and to easier detect e.g. a primary auditory response in the data, we set the low-pass filter to a lower value (20 Hz) (see Fig. 2). Then, the trials were cleaned by the amplitude-based data cleaning procedure described in Methods (EEG artifact rejection and analysis). Mean number of trials were 40.11 in the WORDS (min = 25, max = 67), 40.53 in the SIMILAR (min = 26, max = 72) and 39.12 (min = 23, max = 70) in the NONSENSE condition.

Wilcoxon-signed rank tests

When significant differences between conditions were found in a time-window by repeated-measures ANOVA, we also tested the significant condition-differences by a non-parametric statistical test, Wilcoxon-signed rank test. This test operates on the rank of differences between conditions, hence, the relative magnitude of the condition differences does not influence the results. When differences are also significant by this non-parametric test, it suggests that condition differences revealed by ANOVA are perhaps not only due to some extreme values of individual dogs' data. However, this test might be liberal for normally distributed data.

When the repeated-measures ANOVA did not show any interaction effect between channels and condition in a time-window, Wilcoxon signed-rank test was applied on the mean of Fz and Cz. When there was an interaction effect and the post-hoc tests showed an effect only at one of the electrodes, Wilcoxon signed-rank test was calculated for the values of this one electrode.

Wilcoxon signed-rank tests for effect direction across individuals were significant in all time-windows except one (650-800 ms between SIMILAR and NONSENSE at the data cleaned in three steps) where the effect just barely did not reach significance ($p=0.051$) (Table S2).

Time-window	Condition-pair	#Same	V	P
		Multi-level data cleaning		
650-750	WORDS vs. NONSENSE	12	121	**.035**
650-800	SIMILAR vs. NONSENSE	12	118	.051
		Amplitude-based data-cleaning		
200-300	WORDS vs. NONSENSE	12	122	**.031**
200-300	SIMILAR vs. NONSENSE	12	128	**.013**
650-800	WORDS vs. NONSENSE	13	133	**.006**
700-800*	SIMILAR vs. NONSENSE	11	121	**.035**

Table S2. Results of the Wilcoxon signed-rank test in the selected time-windows for the data cleaned with multi-level and with amplitude-based artifact rejection. The third column shows the number of dogs (out of all 17) showing differences between conditions in the same direction as the significant effect.

* In this time-window, Wilcoxon signed-rank test was calculated on the values at Fz electrode, while all other tests were calculated on the mean of Fz and Cz.

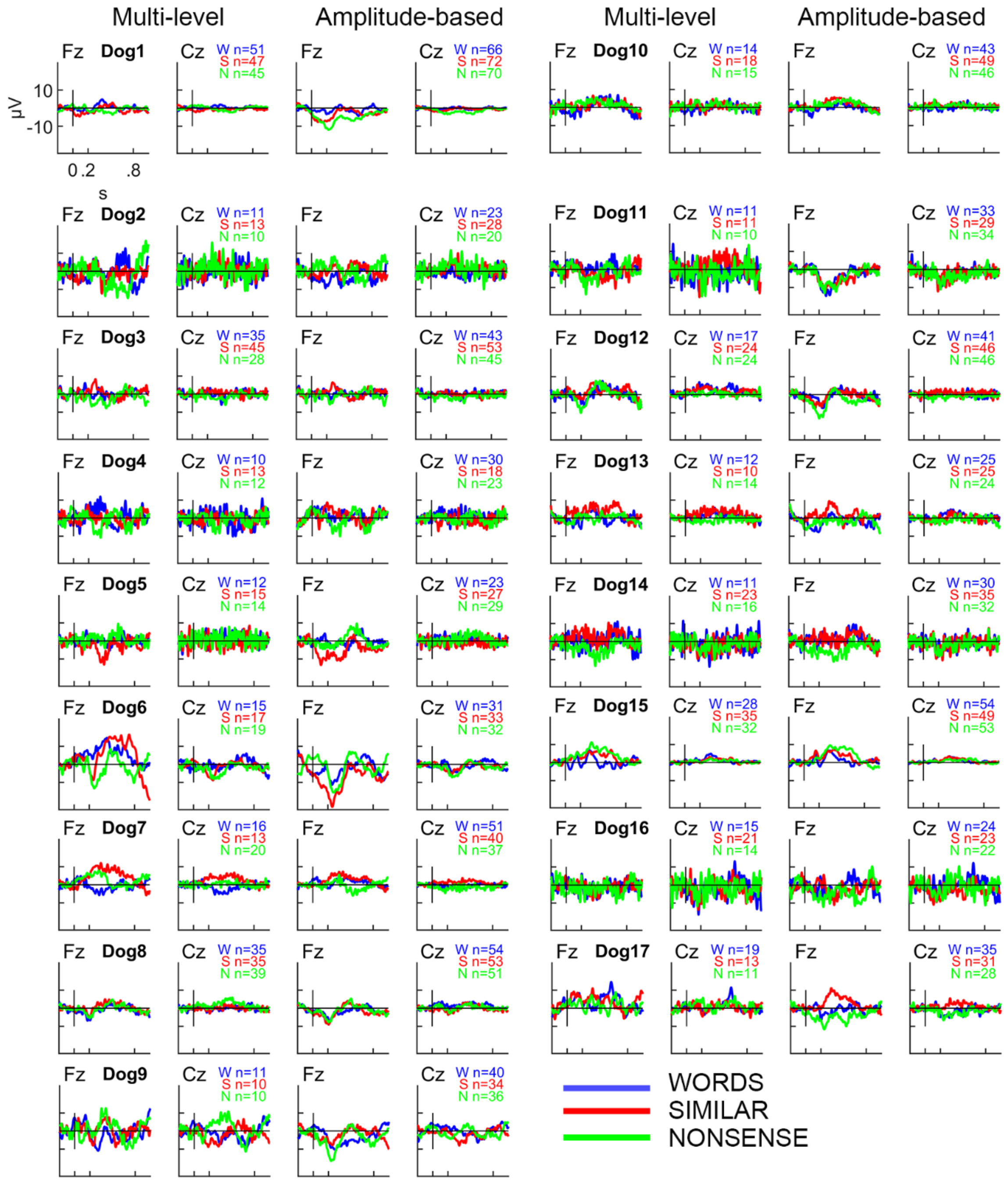

Fig. S2. Event-related potentials (y-axis) for each dog (rows) and condition at Fz and at Cz from -0.2 s to 1 s (x-axis) after the multi-level and the amplitude-based cleaning. Number of trials are shown with color codes for each condition. ERP for WORDS displayed in blue, for SIMILAR in red, for NONSENSE in green.

References:

1. Oravecz, Cs., Váradi, T., & Sass, B. (2014), The Hungarian Gigaword Corpus. In *Proceedings of LREC* 2014.
